# Antimicrobial and Hemolytic Studies of a Series of Polycations Bearing Quaternary Ammonium Moieties: Structural and Topological Effects

**DOI:** 10.3390/ijms18020303

**Published:** 2017-01-30

**Authors:** Judith Mayr, Jürgen Bachl, Jens Schlossmann, David Díaz Díaz

**Affiliations:** 1 Institut für Organische Chemie, Universität Regensburg, Universitätsstr. 31, Regensburg 93053, Germany; judith.mayr@chemie.uni-regensburg.de (J.M.); bachl_j@web.de (J.B.); 2 Institut für Pharmazie, Pharmakologie und Toxikologie, Universität Regensburg, Universitätsstr. 31, Regensburg 93053, Germany; 3 Institute of Advanced Chemistry of Catalonia-Spanish National Research Council (IQAC-CSIC), Jordi Girona 18-26, Barcelona 08034, Spain

**Keywords:** antimicrobial polymer, ionenes, polycations, quaternary ammonium, hemolysis, topomers

## Abstract

A series of polycations bearing quaternary ammonium moieties have shown antimicrobial activity against the Gram-negative bacterium *Escherichia coli*. Different polymer topologies governed by a disubstituted aromatic core as well as different diamine-based linkers were found to influence the antimicrobial properties. Moreover, the hemolytic activity against human red blood cells was measured and demonstrated good biocompatibility and selectivity of these polycations for bacteria over mammalian cells.

## 1. Introduction

Around one fourth of the global deaths annually are caused by infections [1,2,3]. The unjustifiable use of antibiotics and disin–fectants has caused a huge rise in the occurrence of resistant strains [4,5,6,7]. In the USA alone, over 2 million people are infected with these strains and more than 23,000 die yearly for this reason [8]. In Europe more than 150,000 patients are affected by methicillin-resistant *Staphylococcus aureus* (MRSA) infections, which leads to costs of 380 million euros per year [9,10]. Hence, the demand for new antibiotics to combat resistant strains is currently very high. Low molecular weight compounds like antimicrobial peptides have been found to be effective against a wide spectrum of bacteria [11,12]. However, they often comprise disadvantages such as the need for a multistep synthesis or biocidal diffusion, which can cause toxicity and may lead to a fast resistance [13,14]. An alternative and versatile approach is the use of antimicrobial active polymers, whose physical, chemical and biological activities can be fine-tuned by simple modifications of the corresponding monomers. Among different antimicrobial polymers [15,16,17,18,19], ionenes are synthetic polycations with quaternary ammonium functions, which are distributed along the backbone [20,21]. In general, the synthesis of ionenes can be performed by (a) self-polyaddition of aminoalkyl halides, (b) Menshutkin reaction between bis-tertiary amines and activated dihalide compounds or (c) via cationic functionalization of precursor polymers [22,23]. In 1935, Domagk reported for the first time the antibiotic activity of quaternary ammonium salts (QAS) [24]. Since then, different QAS have been extensively investigated as disinfectants [25,26,27,28,29]. Recently, Xiao and co-workers have reported a comprehensive overview of the synthetic methods and antimicrobial action of polymers with quaternary ammonium/phosphonium salts [30]. Their long review demonstrates the high relevance of such materials for the search for new antimicrobial compounds. Related to the type of ionenes that we describe in this paper, Mathias and coworkers reported bis-quaternary ammonium carboxylate polymers based on 1,4-diazabicyclo-[2.2.2]-octane (DABCO) with good activities against *Staphylococcus aureus* and *Escherichia coli* [18]. In addition, Melkonian and her group functionalized cotton to give an antimicrobial material where DABCO was also used to introduce the quaternary ammonium function by attaching different aliphatic chains [31]. Most of these polymers were active against a range of Gram-negative and Gram-positive bacteria.

**Figure 1:**
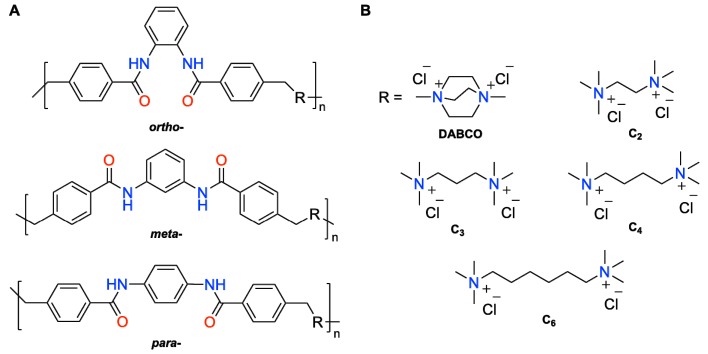
(**A**) General structures of ionene polymers used in this study and (**B**) charged diammonium moieties with different structures and chain length. The combinations investigated in this work were *o*-DABCO, *o*-C_2_, *o*-C_3_, *o*-C_6_, *m*-DABCO, *m*-C_2_, *m*-C_3_, *m*-C_4_, *m*-C_6_, *p*-DABCO, *p*-C_2_, *p*-C_3_ and *p*-C_4_. Note: *o*- = *ortho*-; *m*- = *meta*-; *p*- = *para*-.

Herein, we report the antimicrobial properties of different ionene polymers based on *N*,*N′*-(*p*-phenylene)dibenzamide and α,ω-tertiary diamines, whose self-assembly properties have been previously described by us [32] and others [33]. In our previous work, we demonstrated a remarkable influence of the substitution pattern on the central benzene ring (i.e., *para*-, *meta*-, *ortho*-) on the hydrogelation [32] and dye uptake properties [34] of some of these polymers. In this study, the antimicrobial activity against the Gram-negative bacterium *Escherichia coli* has been determined for a library of 13 ionenes with different topologies and diamine linkers (Figure 1). As far as we are aware, the effect of the polymer topology on the antimicrobial activity has not yet been reported.

## 2. Results and Discussion

Ionene polymers were synthesized via a two steps process as previously reported [32,33,34]. Briefly, first step involves the amidation of *o*-, *m*- and *p*-phenylenediamine with 4-(chloromethyl)benzoyl chloride in the presence of Et_3_N in CH_2_Cl_2_ to afford the corresponding bis-benzamides in good yields (75%–98%) upon recrystallization. Subsequent copolymerization of the obtained bis-benzamides with the desired α,ω-diamine linker under equimolar conditions in dimethylformamide (DMF) at 80 °C afforded white precipitates within 2–6 days. The resulting precipitates were filtered, washed (subsequently with DMF, CH_3_CN and CH_2_Cl_2_) and dried under vacuum to give the desired pure polymers (Figure 1) in modest yields (43%–80%) (Figures S1–S16) [32,33,34]. Although we did not attempt to isolate the products from the supernatant liquid, thin-layer chromatography (TLC) analysis of the reaction crude showed full conversion of the starting materials. Thus, the modest yields obtained during the synthesis of these ionenes could be due to some loss of material during the washing-filtration steps as well as to possible formation of small oligomers with insufficient size to precipitate from the reaction medium. In order to achieve adequate solubility and mobility of the polymers for gel permeation chromatography (GPC) analysis, it was necessary to carry out counteranion exchange of chloride by bis(trifluoromethanesulfonyl)amide (TFSA) anions using lithium bis(trifluoromethanesulfonyl)azanide (LiTFSA) in hot water as previously described [32,33,34]. As expected for many step-growth polymers, these ionenes·TFSA are generally characterized by low degree of polymerizations (*n* = 7–14) and high dispersity values (*Đ* = 2.1–5.7) [32,33,34].

**Figure 2:**
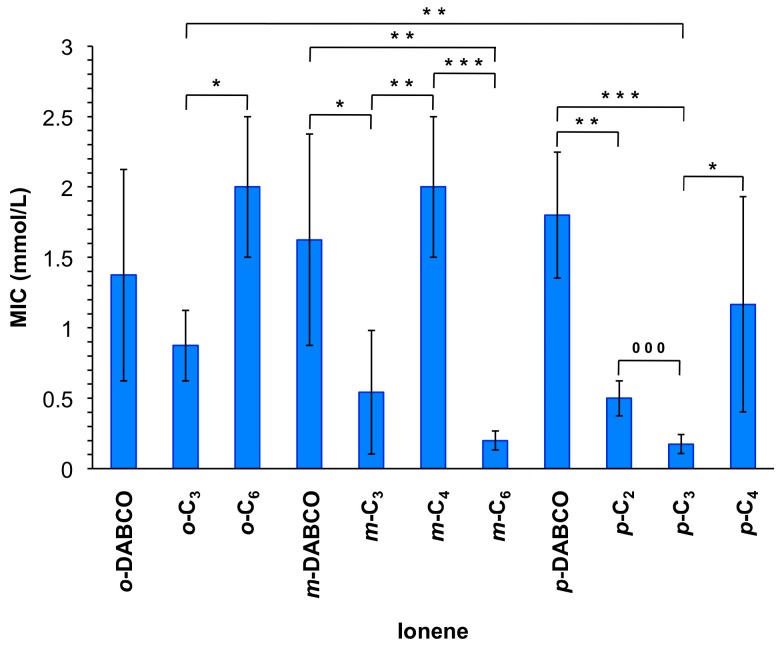
Minimum inhibitory concentrations (MIC) of ionene suspensions for inhibition of bacterial growth. Each value represents the mean of MIC from at least three replicates with standard deviation (SD) and analysis of variances (ANOVA) with Bonferroni comparison or unpaired *t*-test. * Indicates *p* < 0.05; ** indicates *p* < 0.01; ***/^000^ indicates *p* < 0.001. Note: The reported average molarities include, in some cases, chains with a variable number of cationic groups (*vide infra*). For the MIC values expressed in µg/mL see Figure S17.

The resulting ionenes were tested for antimicrobial activity and the minimum inhibitory concentration (MIC) was investigated in a range of 0.125 to 2 mmol/L, respectively (Figure 2). The lowest MIC was found for *m*-C_6_ and *p*-C_3_ with values of 0.2 mmol/L followed by *m*-C_3_ and *p*-C_2_ with MICs of 0.5 and *p*-C_4_ of 1.2 mmol/L. Interestingly, compounds containing DABCO instead of linear linkers showed quite high MICs (i.e., 1.4, 1.6 and 1.8 mmol/L, respectively), similarly to *o*-C_6_ and *m*-C_4_ that revealed MICs of 2 mmol/L. Surprisingly, ionenes *o*-C_2_ and *m*-C_2_ did not show any antimicrobial activity within the measured range. Overall, these results suggested a synergistic role, at least to some extent, between the polymer topology and the nature of the tertiary diamine linker.

Despite the difficulty to draw correlations between the structural moieties and efficiency in bacterial toxicity, DABCO-containing compounds always showed comparably high MIC values (1.4–1.8 mmol/L), only exceeded by *o*-C_6_ and *m*-C_4_ within the experimental error. Interestingly, *o*- and *m*-ionenes bearing the C_2_-linker did not show any antimicrobial activity. This phenomenon could be explained by a judicious degree of flexibility in the polymer chain. Moreover, *o*-substituted polymers also revealed comparably higher MICs (0.8–2.0 mmol/L). The high tendency of these polymers to form coils, which was described in a previous publication [32], could hinder the efficient interaction of these polymers with the cell membrane. Ionenes with the C_3_-linker revealed comparably low or the lowest MICs (0.9, 0.5 and 0.2 mmol/L for the corresponding *o*-, *m*-, and *p*-topomers, respectively). Apparently, a medium sized chain length (C_3_) of the linker in these polymers has the optimal effect on the antimicrobial activity. In contrast, in the case of *m*-topomers, the ionene with the longest chain showed the strongest activity (0.2 mmol/L). It seems evident that a proper balance between flexibility and topological features of these polymers is needed for optimal effects. In comparison to other antimicrobial polymers, our compounds lie in a good middle field. As representative examples, Tew and co-workers described quaternary pyridinium functionalized polynorbornenes with MICs ranging from 4–200 µg/mL (7.2 × 10^−3^–0.40 mmol/L) [3]; Lecomte’s group described a poly(oxepan-2-one) with quaternary ammonium with a very high MIC of 12.6 mg/mL (23 mmol/L) [15] and Mathias and coworkers reported bis-quaternary ammonium methacrylate polymers with MICs ranging from 62.5 to 250 µg/mL (0.095–0.40 mmol/L) [18]. It is worth mentioning that most of the ionenes showed a polymerization degree (*n*) between 7 and 9 (only for *p*-C_2_, *p*-C_3_ and *o*-C_6_ *n* was 12, 13 and 14, respectively; Table S1) and no significant correlations could be drawn between this parameter and the antimicrobial activity. Note that the polymerization degree was calculated for the corresponding TFSA salt of the polymers when the counteranion exchange with LiTFSA was successful as explained above (this was necessary to provide enough solubility for the analysis). These results suggest that much larger differences in *n,* and therefore on the number of cationic units, are necessary to have a clear impact on the antimicrobial activity of these ionenes.

**Figure 3:**
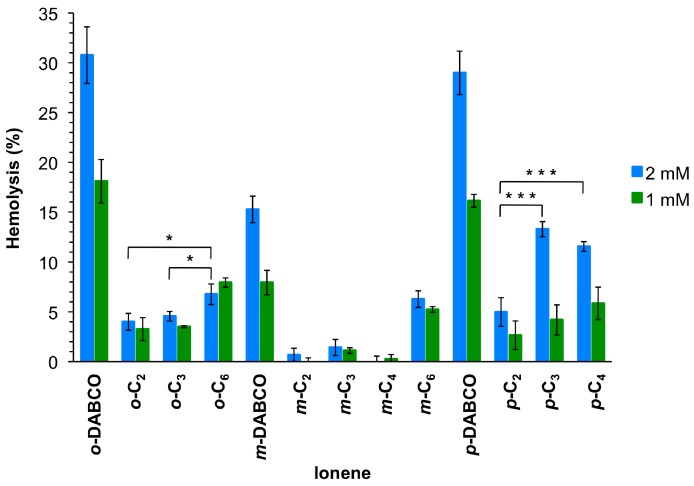
Mean values of hemolysis from at least three replicates with SD and ANOVA with Bonferroni comparison. For the sake of clarity, only comparisons within one topomeric set (*ortho*-, *meta*-, *para*-) at 2 mmol/L are shown in this plot. All DABCO-containing compounds showed higher hemolysis (***, omitted for clarity) in comparison to their set members. * Indicates *p* < 0.05; *** indicates *p* < 0.001. See Supplementary Material for additional comparisons (Figures S19 and S20).

Since antimicrobial agents should be ideally not harmful to mammalian cells, the hemolytic activity of these polymers was also tested at similar concentration as for the antimicrobial assay. Thus, the polymer solutions were measured at 2 mmol/L, which was the limit for antimicrobial studies, and 1 mmol/L for comparison (Figure 3). The results showed that these ionenes are not or only slightly hemolytic, judged by very low levels of released hemoglobin in most cases. However, release of hemoglobin increased with increasing polymer concentration. In general, DABCO-containing polymers revealed the highest hemoglobin levels, being *m*-DABCO the ionene of this class with the lowest values (15% and 8% for 2 and 1 mmol/L, respectively). About 10% reduction of hemoglobin release was observed for *m*-DABCO compared to *o*-DABCO and *p*-DABCO at both concentrations. The remaining polymers showed hemoglobin release ranging between 13% for *p*-C_3_ 2 mmol/L and 1% for *m*-C_3_ at both concentrations. In general, the length of the linear linker within the same series of topomers showed an irregular effect on the hemolytic properties. Thus, the lowest hemolysis was observed for *p*-and *o*-topomers with a C_2_-linker (5% and 4%, respectively) at 2 mmol/L, whereas the same the C_4_-linker provided the lowest hemoglobin release for the *m*-series (0.25%). On the other hand, the topology of the polymers seemed to be a more important factor at higher concentration (2 mmol/L) within the series bearing the same linker, albeit again without a regular trend. Thus, *m*-topomers displayed lower hemoglobin release than the corresponding *o*- and *p*-topomers with a C_2_-linker (4% vs. 1% and 5%, respectively). The same was found for C_3_-, C_4_- and DABCO-linkers, whereas no significant differences were found for the series based on C_6_-linkers. With respect to the concentration effect, the most pronounced differences between the two concentrations were found for the *p*-series. For comparison, hemolysis obtained with other polycations such as poly(diallyl-dimethyl-ammonium chloride) and poly(vinyl pyridinium bromide) were found to be 2.6% and 9.4%, respectively [35], within the same range of concentration as we used for this investigation.

In contrast to other polycations [36], no obvious correlations were found between the antimicrobial properties or hemolytic activities of most of the ionenes studied in this work and their surface wettability determined by contact angle measurements (Figure S18). Nevertheless, DABCO-containing polymers displayed in general higher MIC, hemolytic activity and higher hydrophilic character (i.e., contact angle <30° for *m*-DABCO and *p*-DABCO) compared to the ionenes bearing linear linkers, other significant correlations among the different linkers and topological features of the polymers were not found. Youngblood and co-workers demonstrated the relationship between antibacterial activities and surface wettability for a series of quaternized polymers made via copolymerization of vinylpyridine with hydroethyl methacrylate and poly(ethylene glycol) methyl ether methacrylate [36]. In addition, the authors demonstrated the superior antimicrobial activity of the copolymers in comparison to the pure poly(vinylpyridine) homopolymer. Unfortunately, the contact angle of the films made from our ionenes based on linear linkers did not follow any pattern for that could be correlated to the observed antibacterial properties (Figure S17). Maybe the higher dispersity of these polymers compared to those of poly(vinylpyridine) copolymers may hamper to establish such correlations. However, the results obtained from DABCO-based ionenes suggest that the introduction of more hydrophilic domains into the structures of this type of ionenes could enhance their antimicrobial activities. Probably, the higher rigidity of DABCO compared to the other linear aliphatic linkers could minimize conformational changes in those polymers [32], allowing to observe more clearly the effect caused by their topologies.

## 3. Materials and Methods

### 3.1. Materials

Starting materials for polymer synthesis were purchased from Sigma Aldrich (St. Louis, MO, USA) or TCI (Zwijndrecht, Belgium). TGYE-medium ingredients were from Becton, Dickinson and Company (Franklin Lakes, NJ, USA). Müller-Hinton-Broth was purchased from Merck (Darmstadt, Germany). Hemolysis was measured on a sunrise tecan microplate reader (Tecan, Männedorf, Switzerland). All ionene polymers were synthesized following the procedure previously described and showed the same spectroscopic data to those reported therein [33,34].

### 3.2. Antimicrobial Assay

TGYE-medium (30 mL) was inoculated with one colony of *Escherichia coli*, incubated at 37 °C while orbital shaking at 150 rpm overnight. From this suspension, a 1:100 dilution was prepared in Müller-Hinton-Broth (MHB) and 50 µL of this were added to 1 mL of solutions/dispersions of the polymers at different concentrations in MHB (i.e., 2, 1, 0.5, 0.25 and 0.125 mM from serial dilution starting from 4 mM polymer in sodium chloride 9 g/L solution). The bacteria were incubated at 37 °C for 18 h. After this time 50 µL of sample were spread on Trypton-Glucose-Yeast (TGYE) agar plates, which were incubated at 37 °C for 22 h and bacterial growth was optically evaluated. MIC was determined as the lowest concentration of polymers at which no visible growth was observed. For comparative purposes, all results reported in this work are referred to the molar mass of the repeating unit of the corresponding polymer.

### 3.3. Hemolysis Testing

Blood from a healthy human volunteer was drawn into K2-EDTA coated vacutainer tubes and stored at 4 °C for 1 h. The blood was centrifuged at 1000× *g* for 10 min. The erythrocytes were washed with 2 mL PBS buffer and centrifuged again (3 times). The erythrocytes were resuspended in PBS buffer to give a 5% (*v*/*v*) suspension. Solutions/dispersions of polymers in sodium chloride 9 g/L solution (60 µL), erythrocyte suspensions (60 µL) and PBS buffer (60 µL) were mixed. As positive hemolysis control Triton-X100 1% (*v*/*v*) (60 µL) and as negative control PBS buffer (60 µL) was used. Samples were incubated at 37 °C and orbital shaking at 150 rpm for 2 h followed by centrifugation at 1000× *g* for 5 min. Sample solution (60 µL) and PBS buffer (60 µL) were filled into a 96 well plate and absorbance was measured at 540 nm. Hemolysis was calculated by following equation.

(1) $$hemolysis=(\frac{S_{\mathrm{PBS}}-S_{\mathrm{Poly}}}{S_{\mathrm{TX}}-S_{\mathrm{Poly}}})\times 100\%$$

where *S*_PBS_ is the value corresponding to the negative control, *S*_TX_ is the value corresponding to the positive control and *S*_Poly_ is the value corresponding to the polymer sample.

### 3.4. Contact Angle Measurements

Polymer solutions/suspensions were prepared at a concentration of 50 g/L in DMSO/H_2_O (1:1, *v*/*v*). Menzel cover slips (18 mm × 18 mm) were washed with acetone and ethanol before usage. Samples (50 µL) were spread on the glass plates to cover as much as possible of the surface. The plates were left on air for 3 days to dry. The measurements were performed with a Dataphysics Contact Angle System OCA (Dataphysics, Germany) by dropping 3 µL on the surface and images were analyzed with the software SCA 20.

## 4. Conclusions

In summary, ionene polymers with different topologies derived from a disubstituted aromatic monomeric core and α,ω-ditertiary amines were prepared via step-growth polymerization. The polymers showed antimicrobial activity against the Gram-negative bacterium *Escherichia coli*, with MIC values affected by both the topology of the polymer and the nature of the diamine linker. In general, all ionenes containing DABCO (1.4–1.8 mmol/L), as well as *ortho*-compounds with other linkers (0.8–2.0 mmol/L), showed higher MICs. In contrast, medium chain length (C_3_) showed best or comparably low MICs (0.9, 0.5 and 0.2 mmol/L for *ortho*-, *meta*- and *para*-, respectively). In this assay, all DABCO-containing polymers showed a relatively high hemoglobin release (15%–31%) whereas *meta*-compounds in general provided the lowest hemolysis (0%–15%). In conclusion, *meta*-compounds with a flexible linker provided the best properties from both assays, and represent the most promising candidates for potential antimicrobial applications.

## Supplementary Materials

The following are available online at www.mdpi.com/1422-0067/18/2/303/s1.
