# Supplementary material for: Antimicrobial and Hemolytic Studies of a Series of Polycations Bearing Quaternary Ammonium Moieties: Structural and Topological Effects
Source: Int J Mol Sci. 2017 Jan 30;18(2):303. doi: 10.3390/ijms18020303 (PMC5343839; doi:10.3390/ijms18020303)
Supplement: Supplementary file 1 [file ijms-18-00303-s001.docx]

Supplementary Material:
Antimicrobial and Hemolytic Studies of a Series of Polycations Bearing Quaternary Ammonium Moieties: Structural and Topological Effects

Judith Mayr, Jürgen Bachl, Jens Schlossmann and David Díaz Díaz


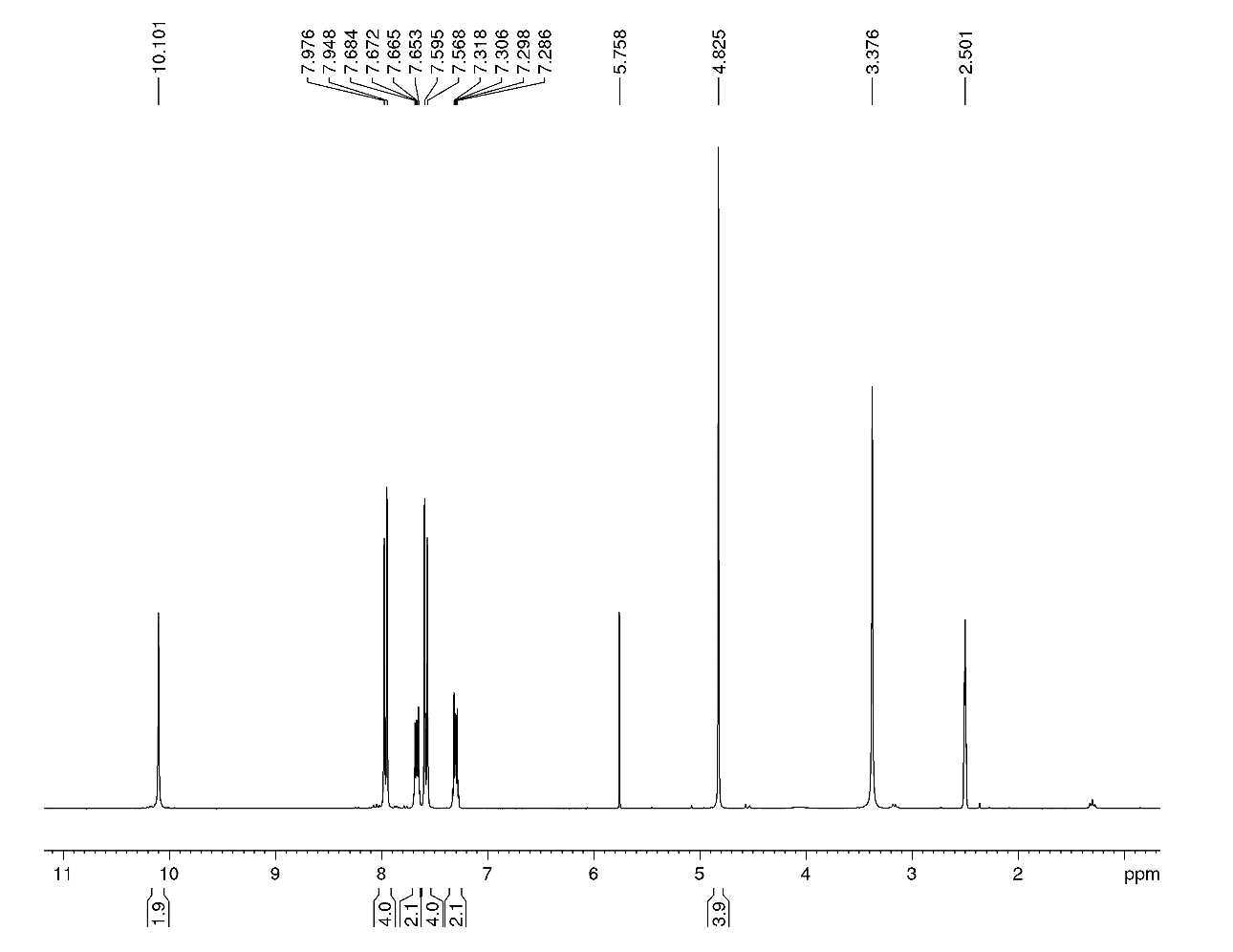


**Figure S1.** ^1^H NMR (300 MHz, DMSO-*d_6_*) of *ortho*-monomer.


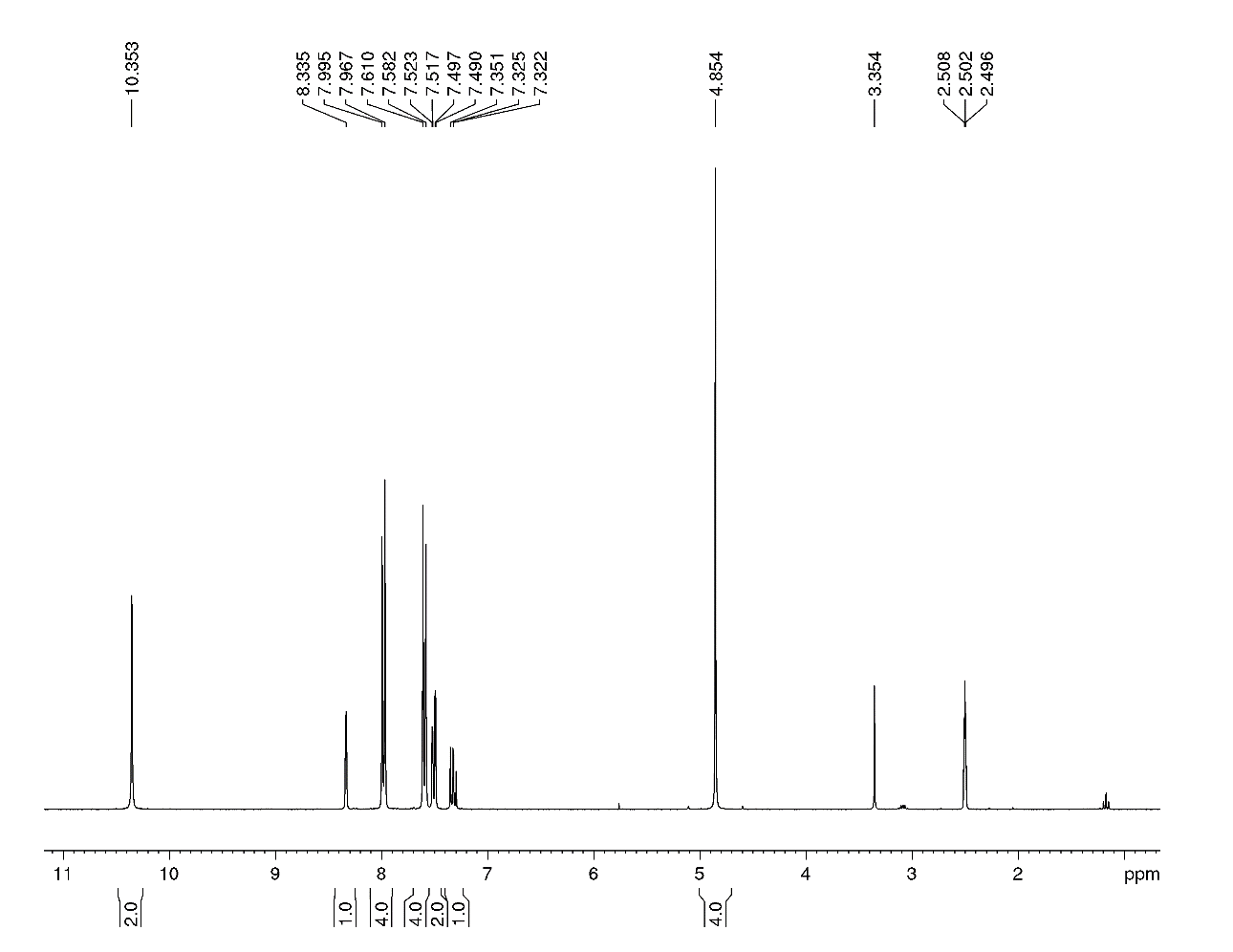


**Figure S2.** ^1^H NMR (300 MHz, DMSO-*d_6_*) of *meta*-monomer.


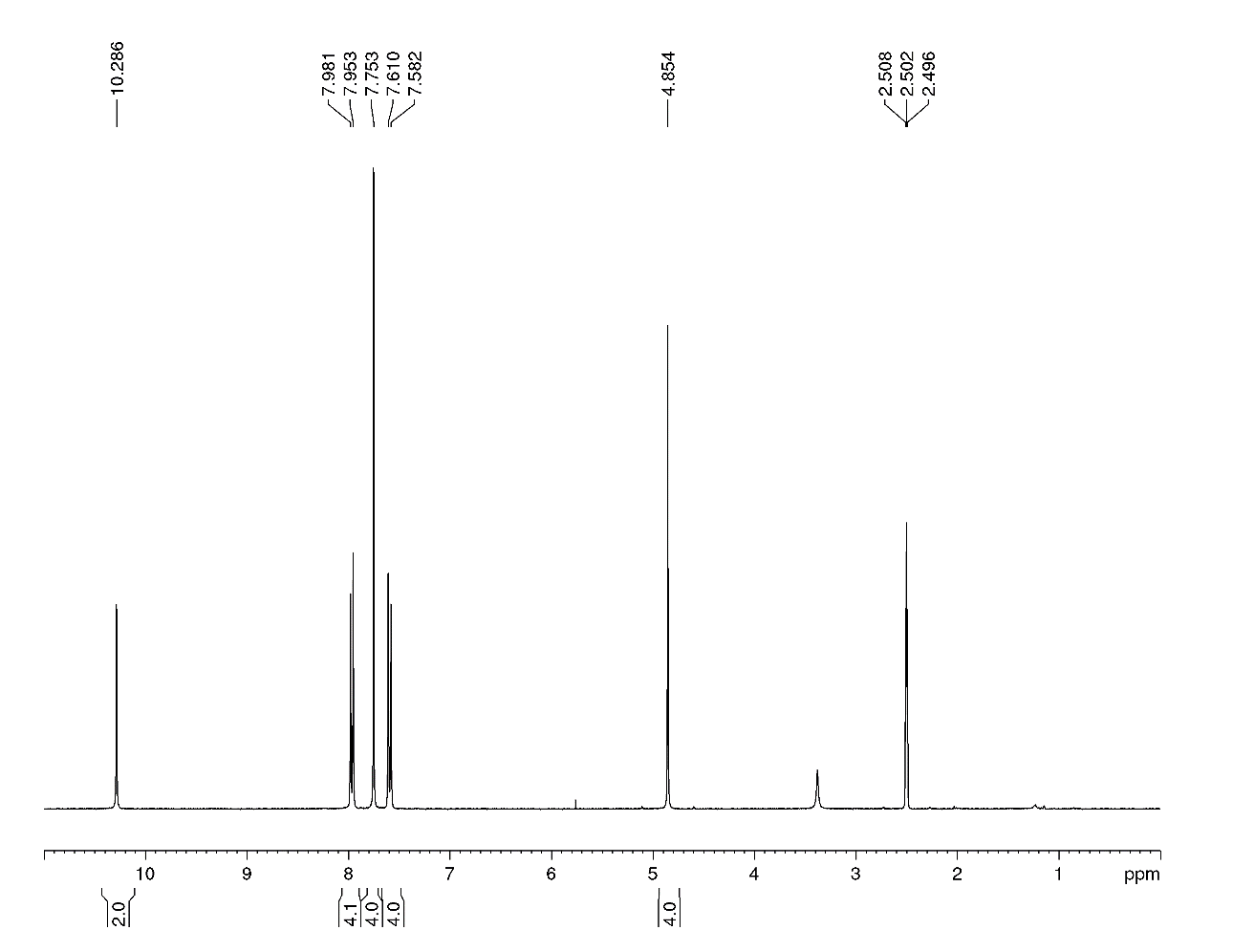


**Figure S3.** ^1^H NMR (300 MHz, DMSO-*d_6_*) of *para*-monomer.


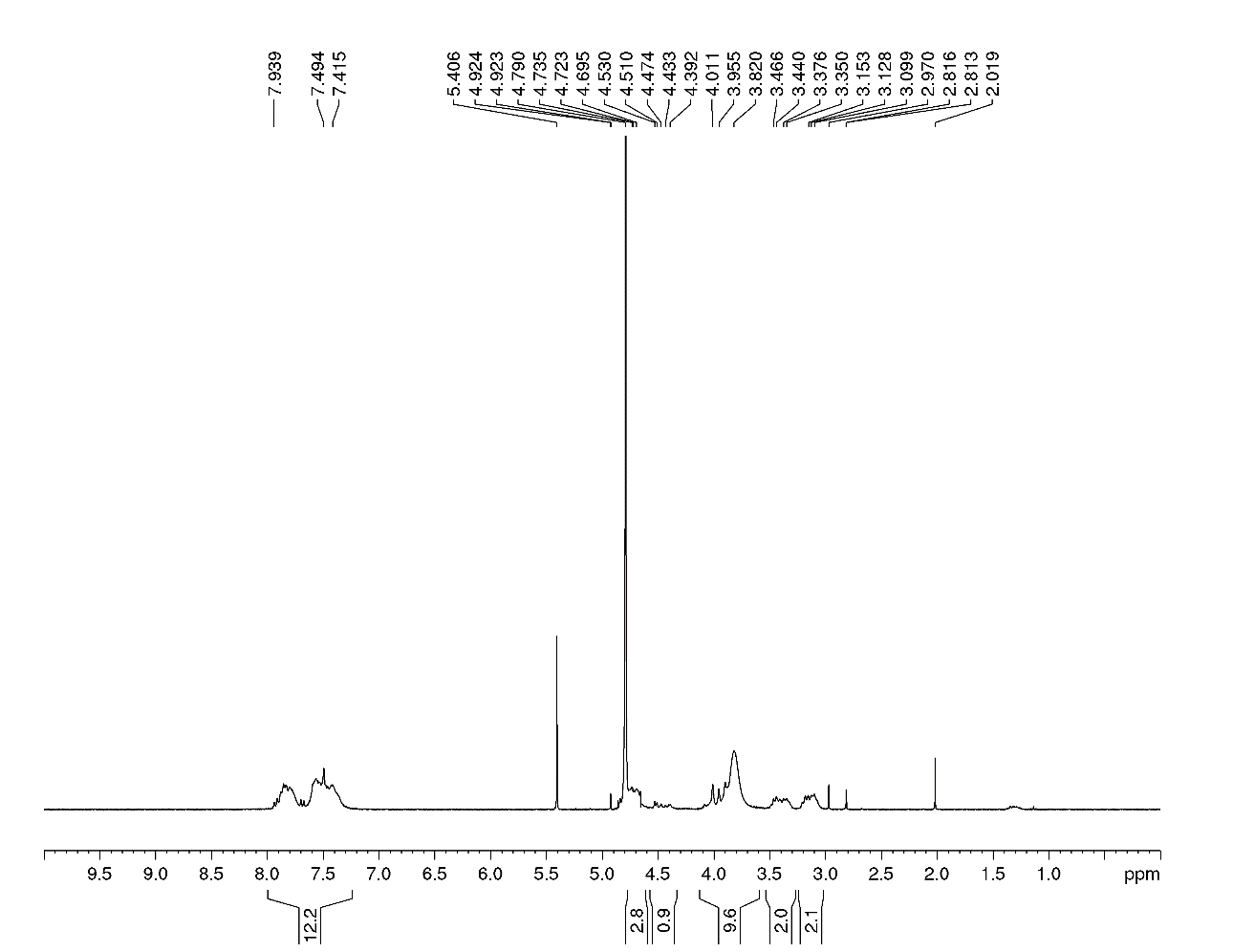


**Figure S4.** ^1^H NMR (300 MHz, D_2_O) of *o*-DABCO.


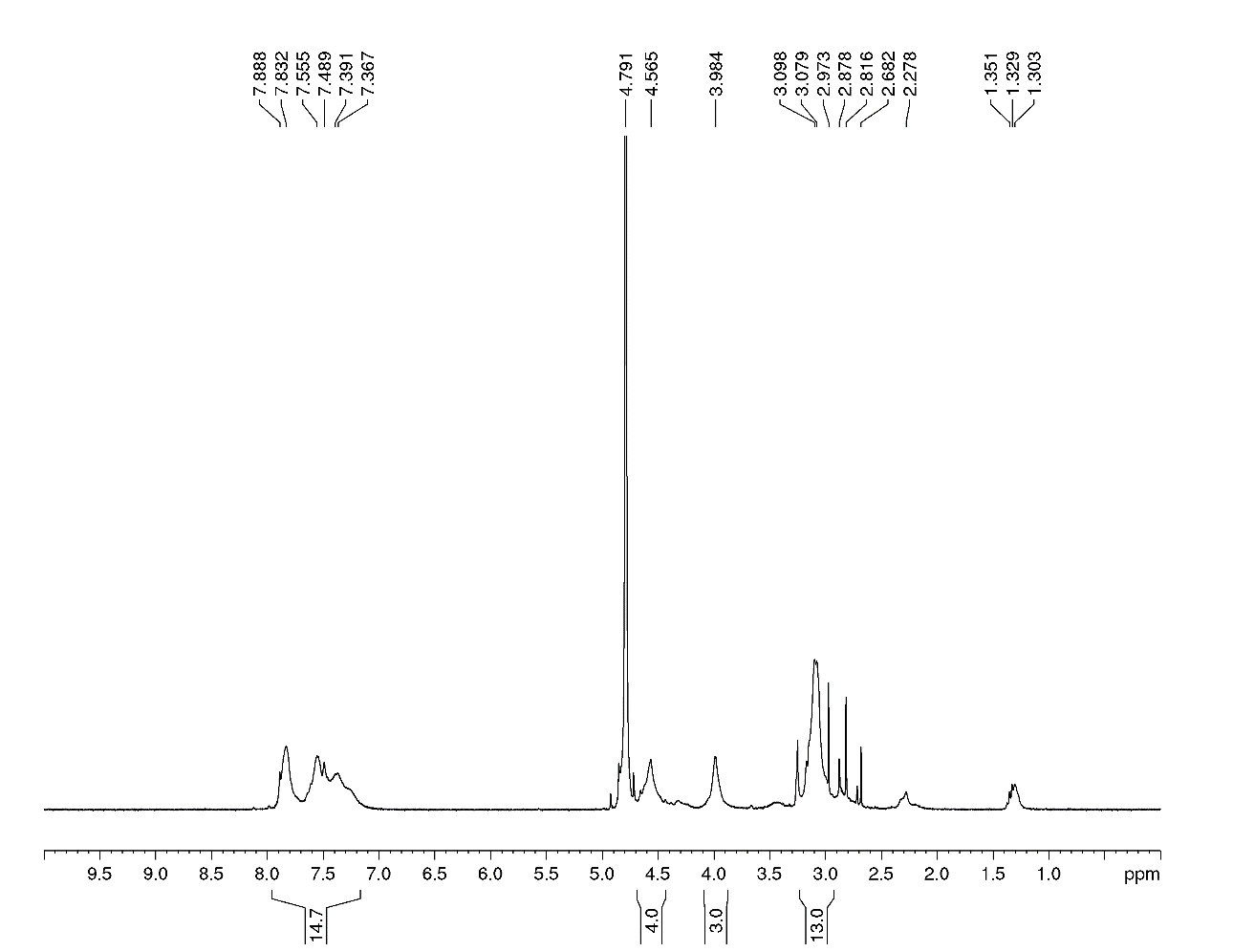


**Figure S5.** ^1^H NMR (300 MHz, D_2_O) of *o*-C_2_.


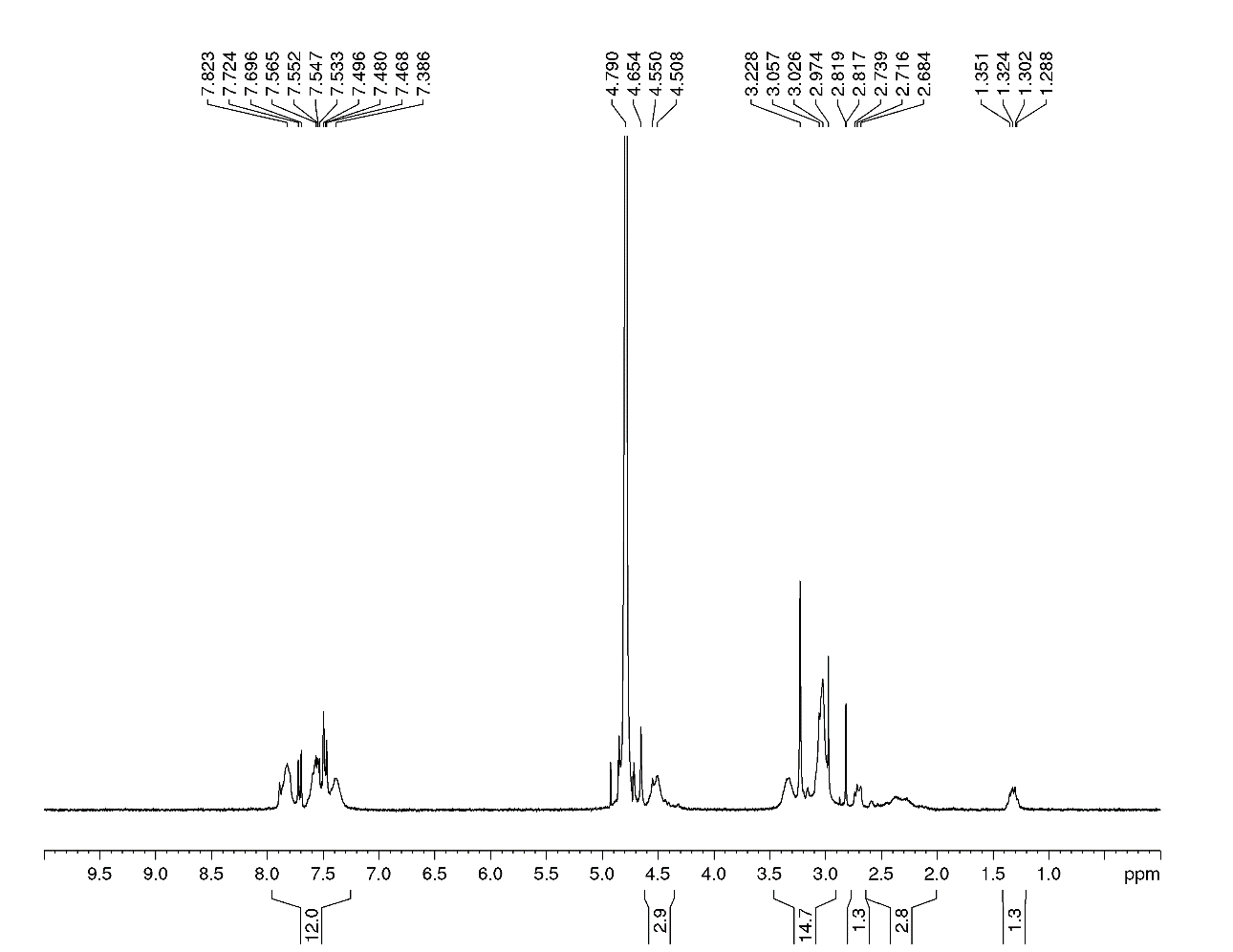


**Figure S6.** ^1^H NMR (300 MHz, D_2_O) of *o*-C_3_.


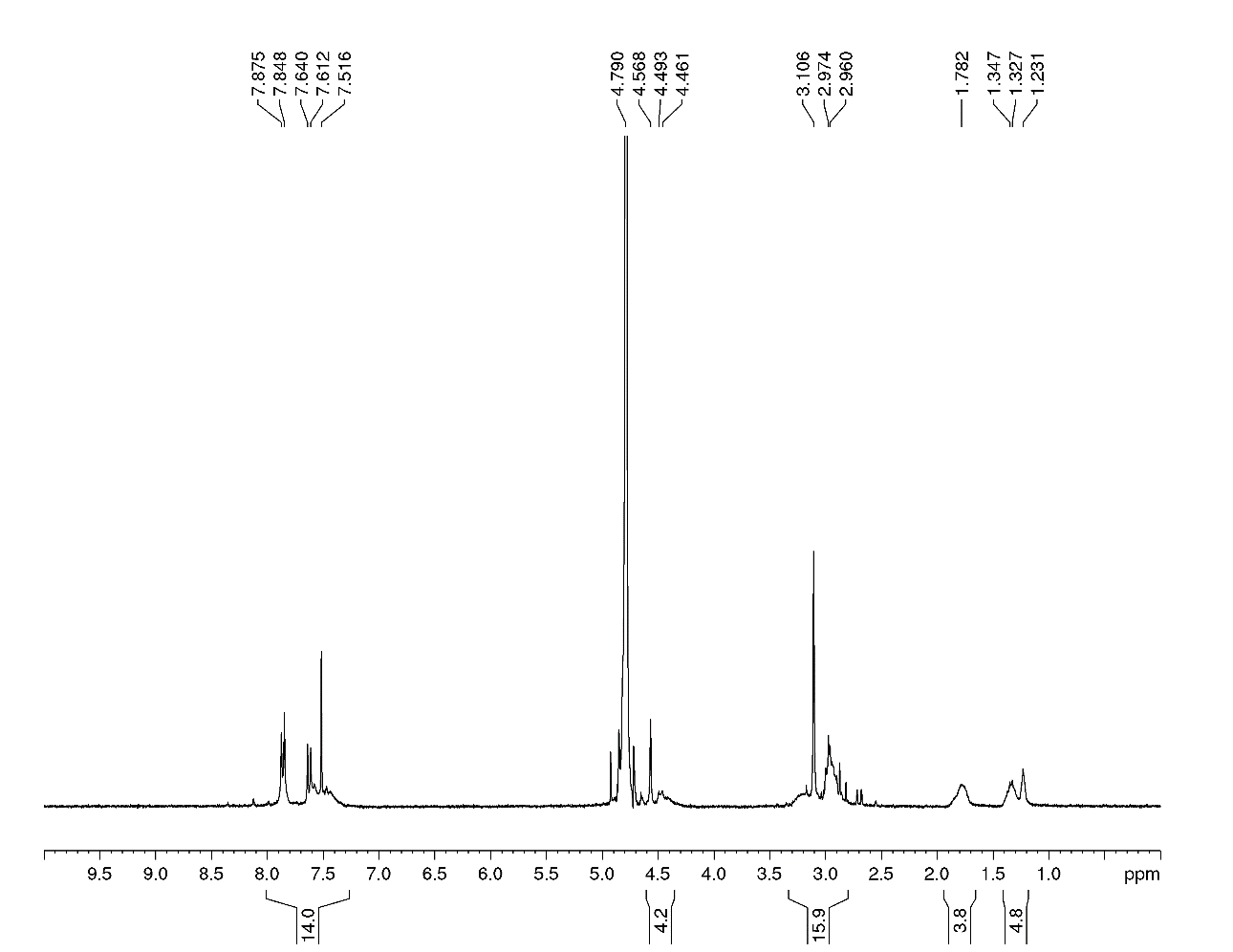


**Figure S7.** ^1^H NMR (300 MHz, D_2_O) of *o*-C_6_.


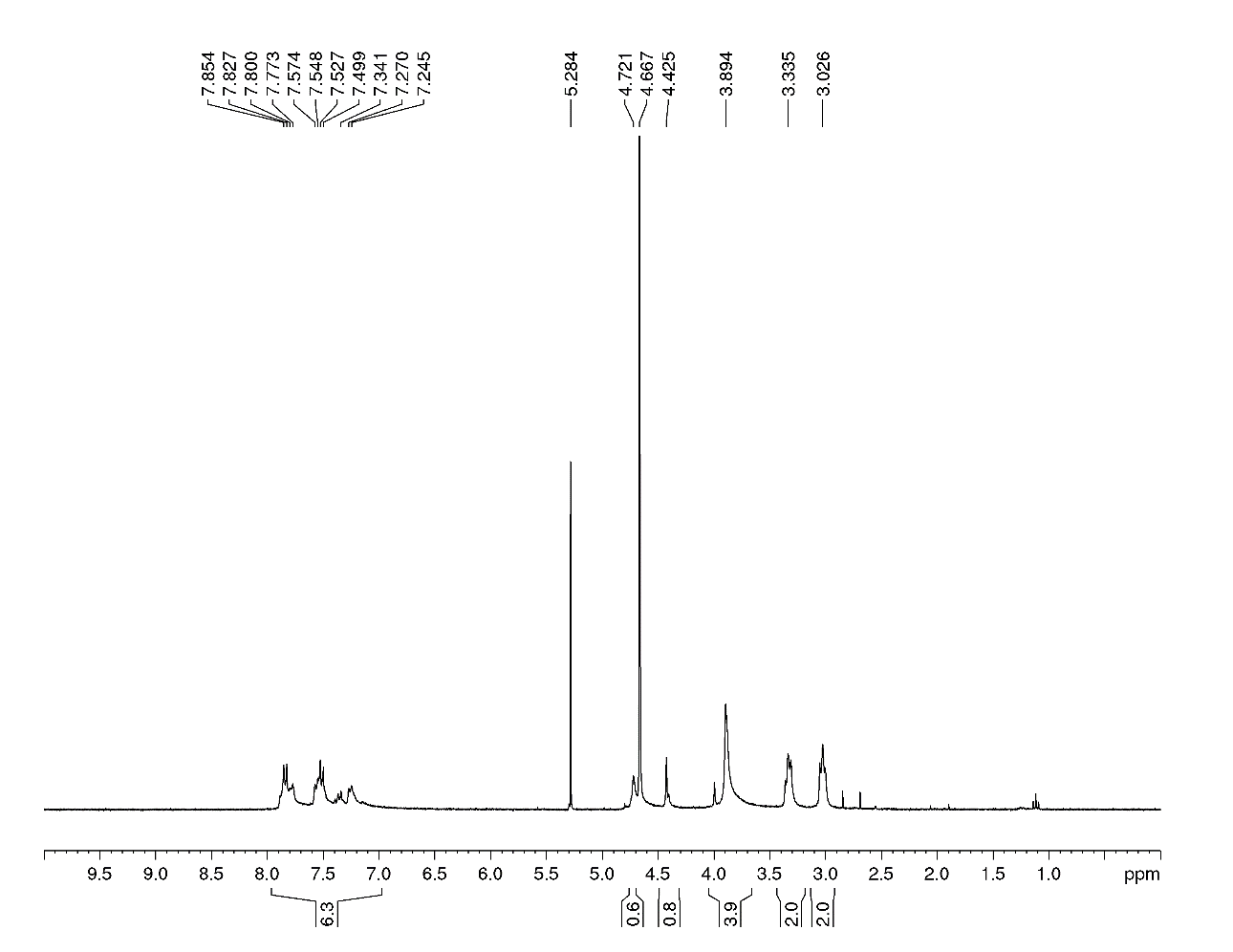


**Figure S8.** ^1^H NMR (300 MHz, D_2_O) of *m*-DABCO.


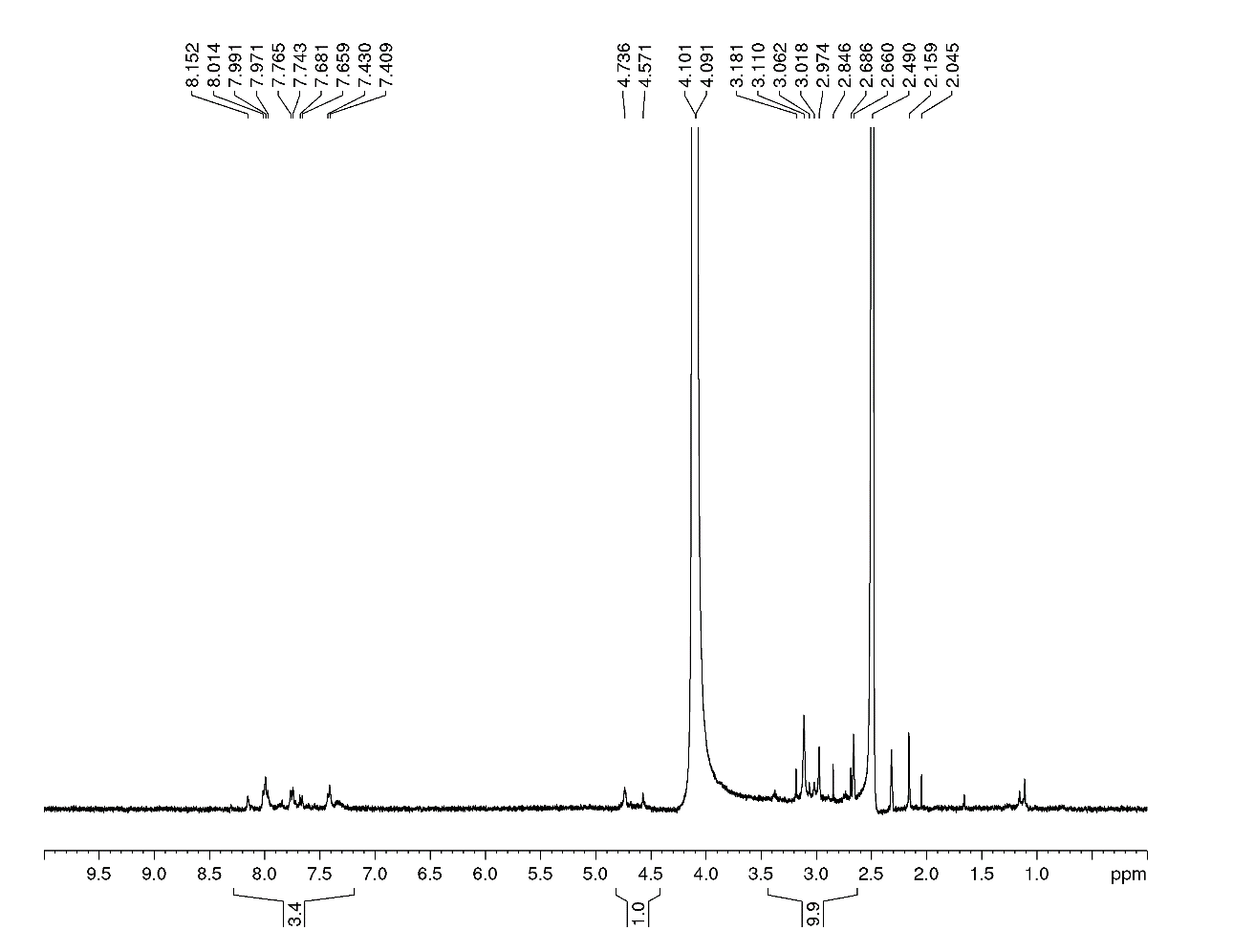


**Figure S9.** ^1^H NMR (400 MHz, DMSO-*d_6_*: D_2_O, 5:1 *v*/*v*) of *m*-C_2_.


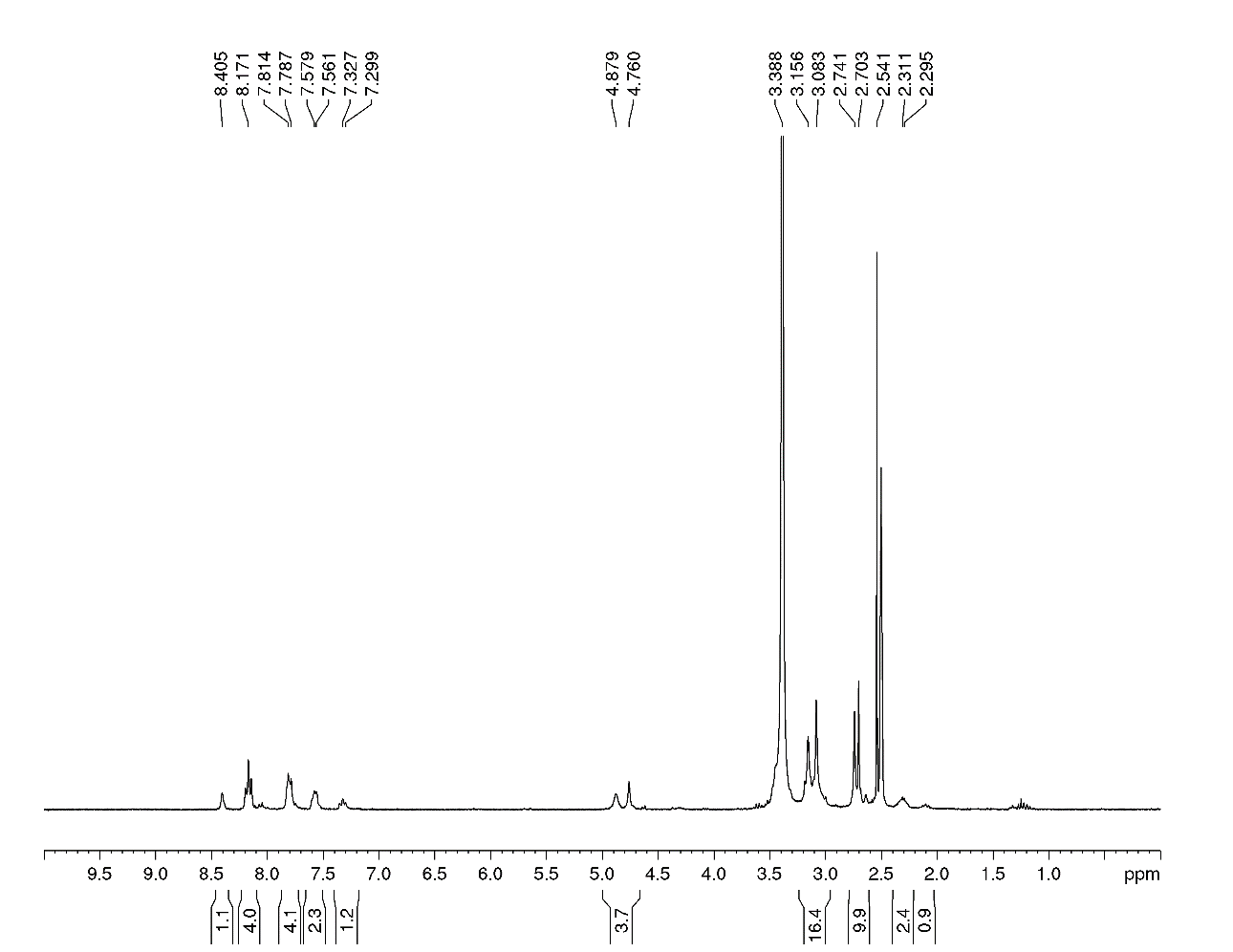


**Figure S10.** ^1^H NMR (300 MHz, D_2_O) of *m*-C_3_.


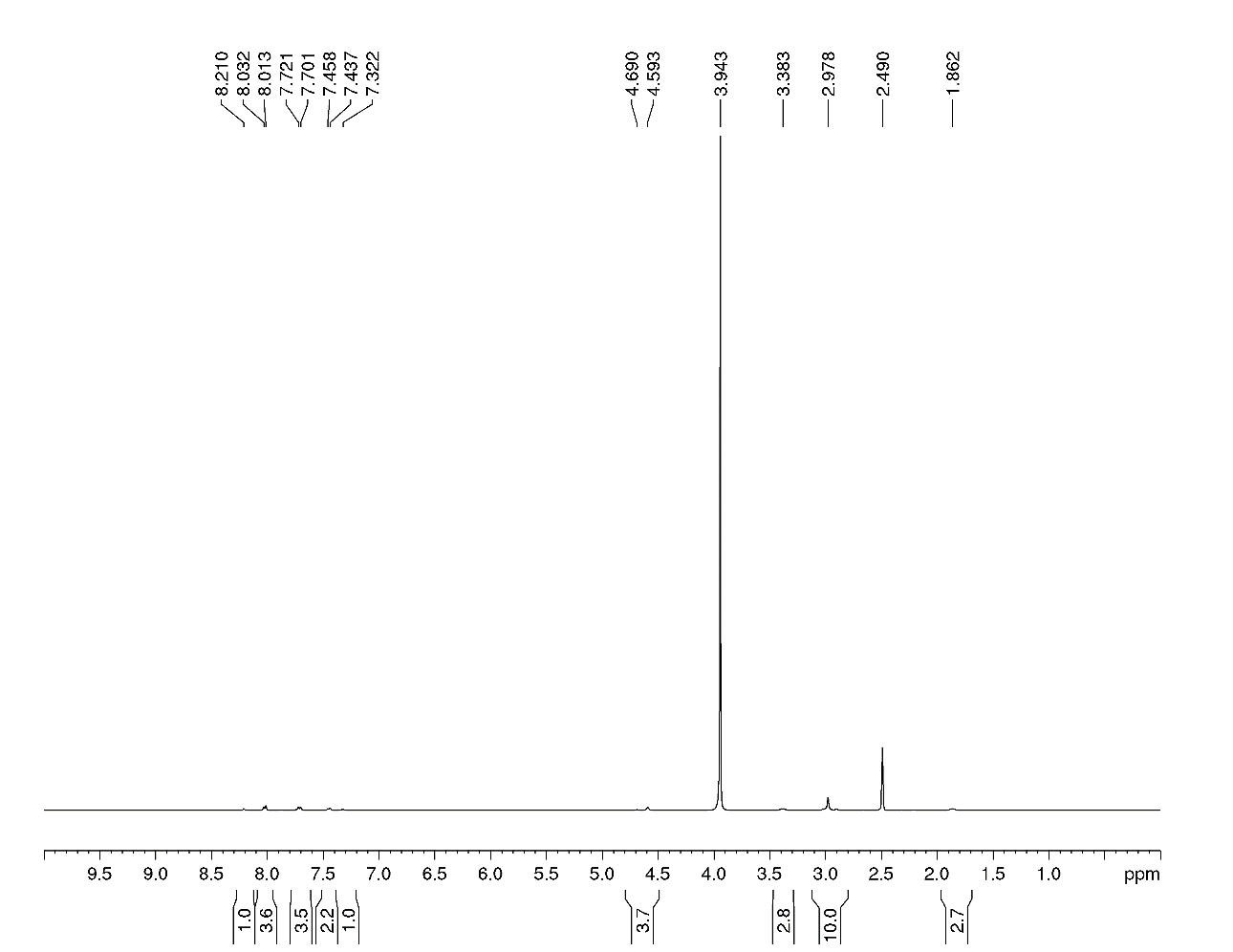


**Figure S11.** ^1^H NMR (400 MHz, DMSO-*d_6_*: D_2_O, 5:1 *v*/*v*) of *m*-C_4_.


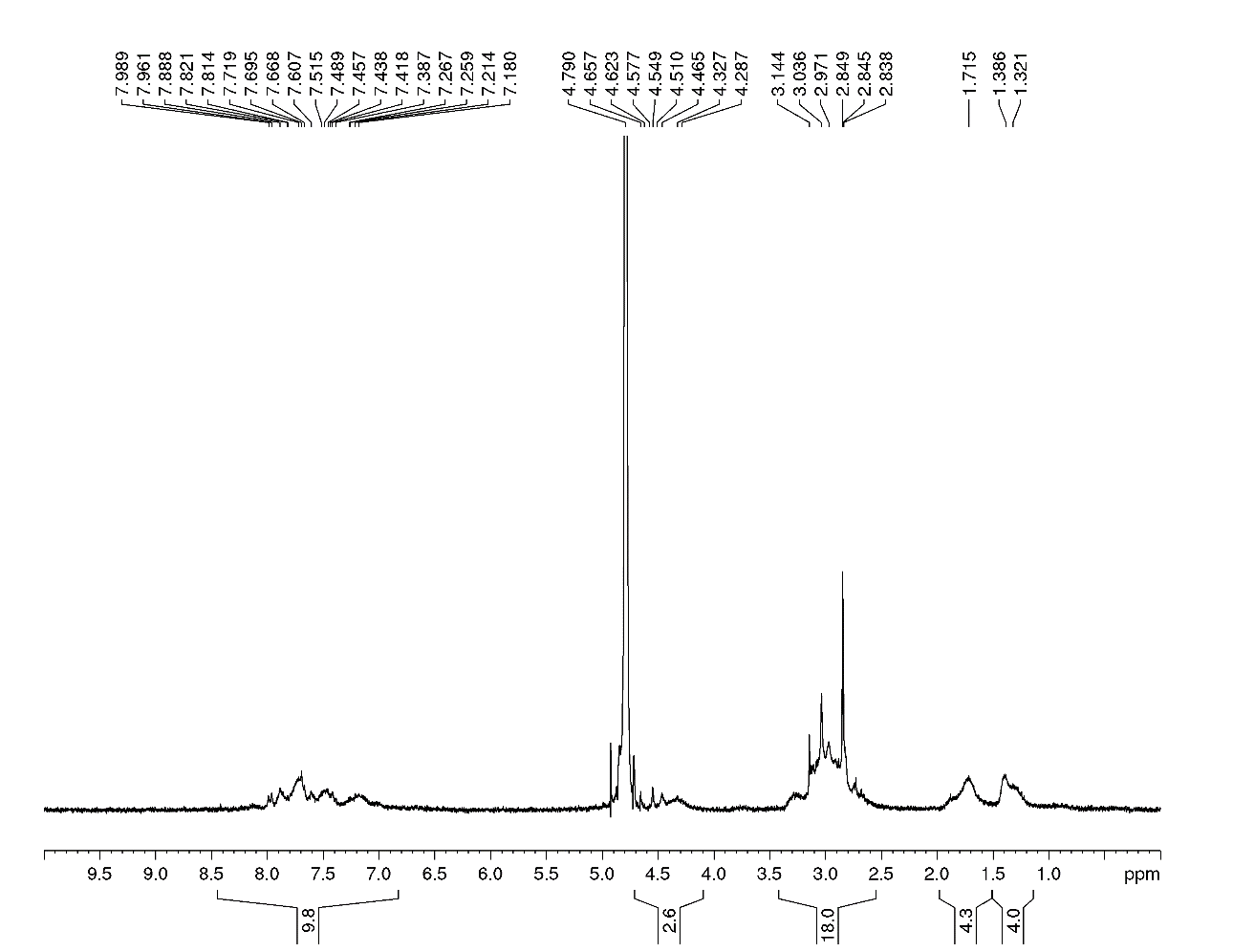


**Figure S12.** ^1^H NMR (300 MHz, D_2_O) of *m*-C_6_.


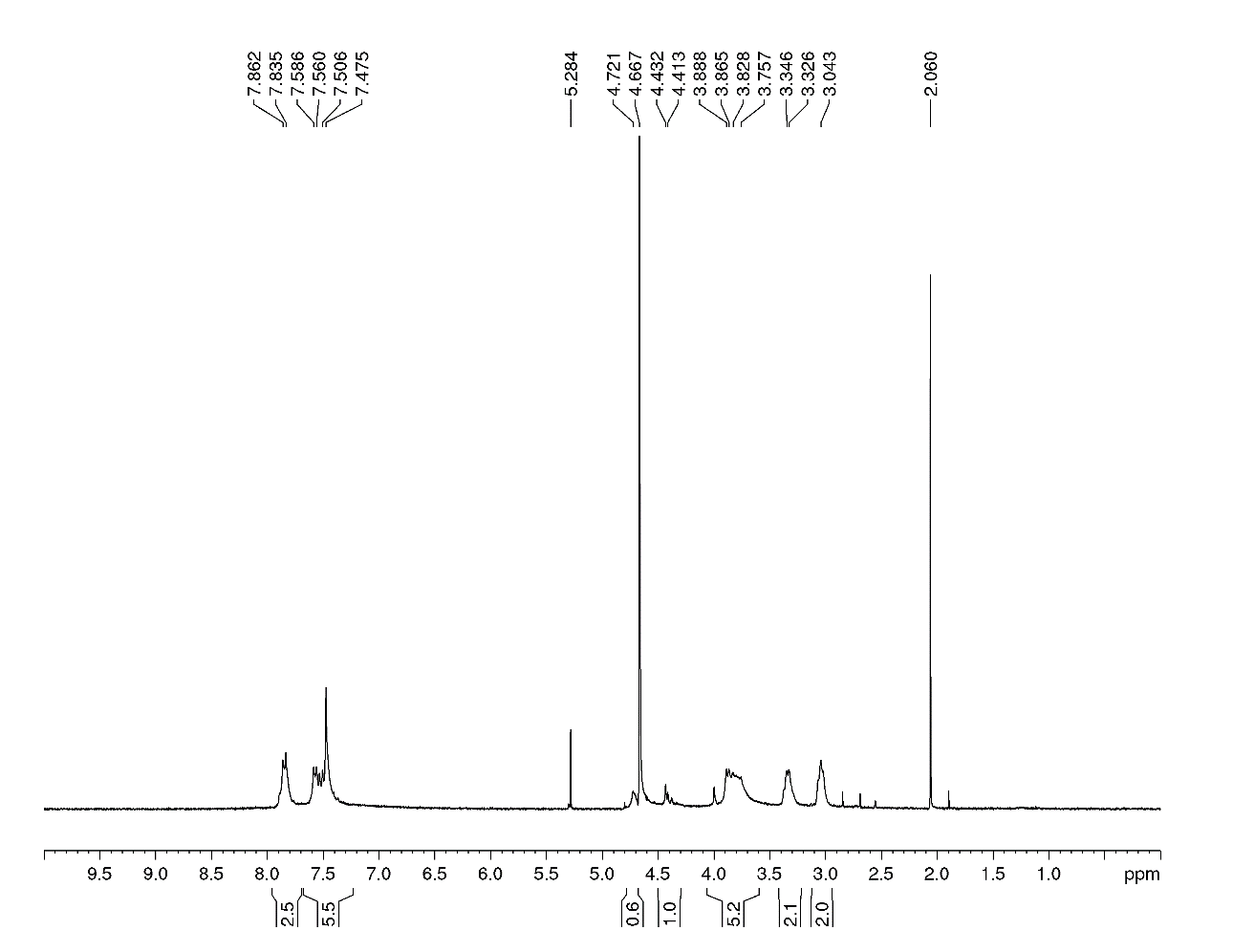


**Figure S13.** ^1^H NMR (300 MHz, D_2_O) of *p*-DABCO.


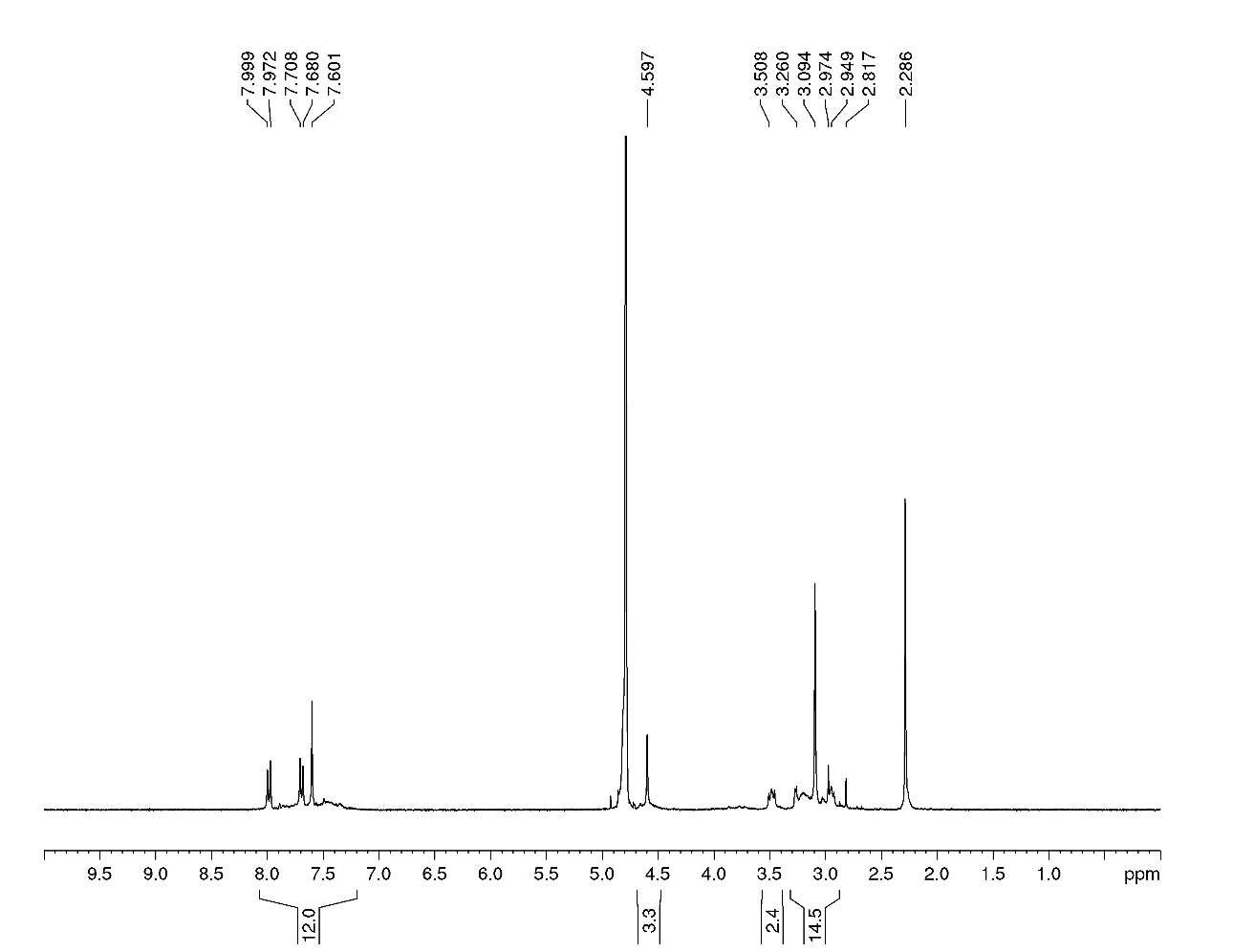


**Figure S14.** ^1^H NMR (300 MHz, D_2_O) of *p*-C_2_.


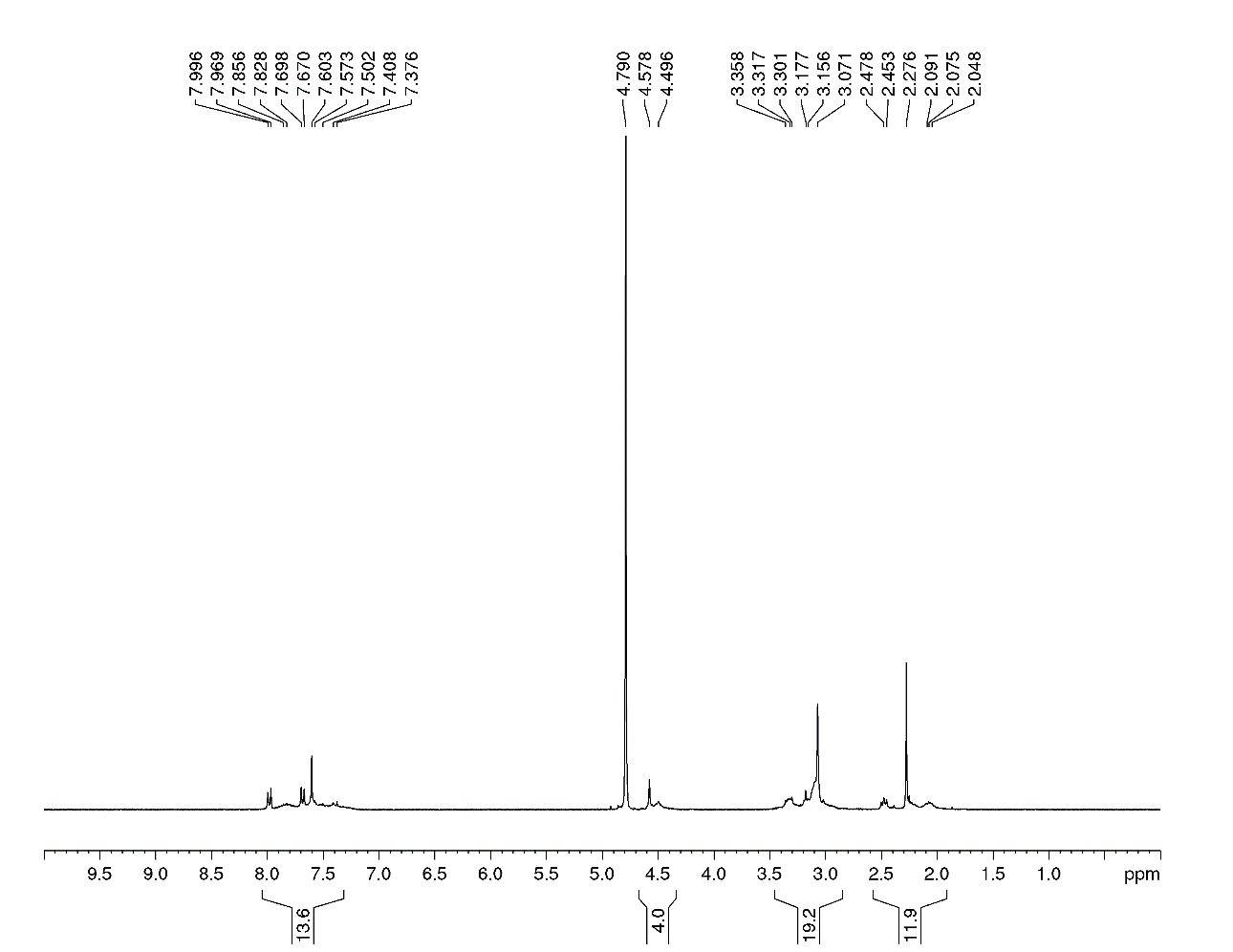


**Figure S15.** ^1^H NMR (300 MHz, D_2_O) of *p*-C_3_.


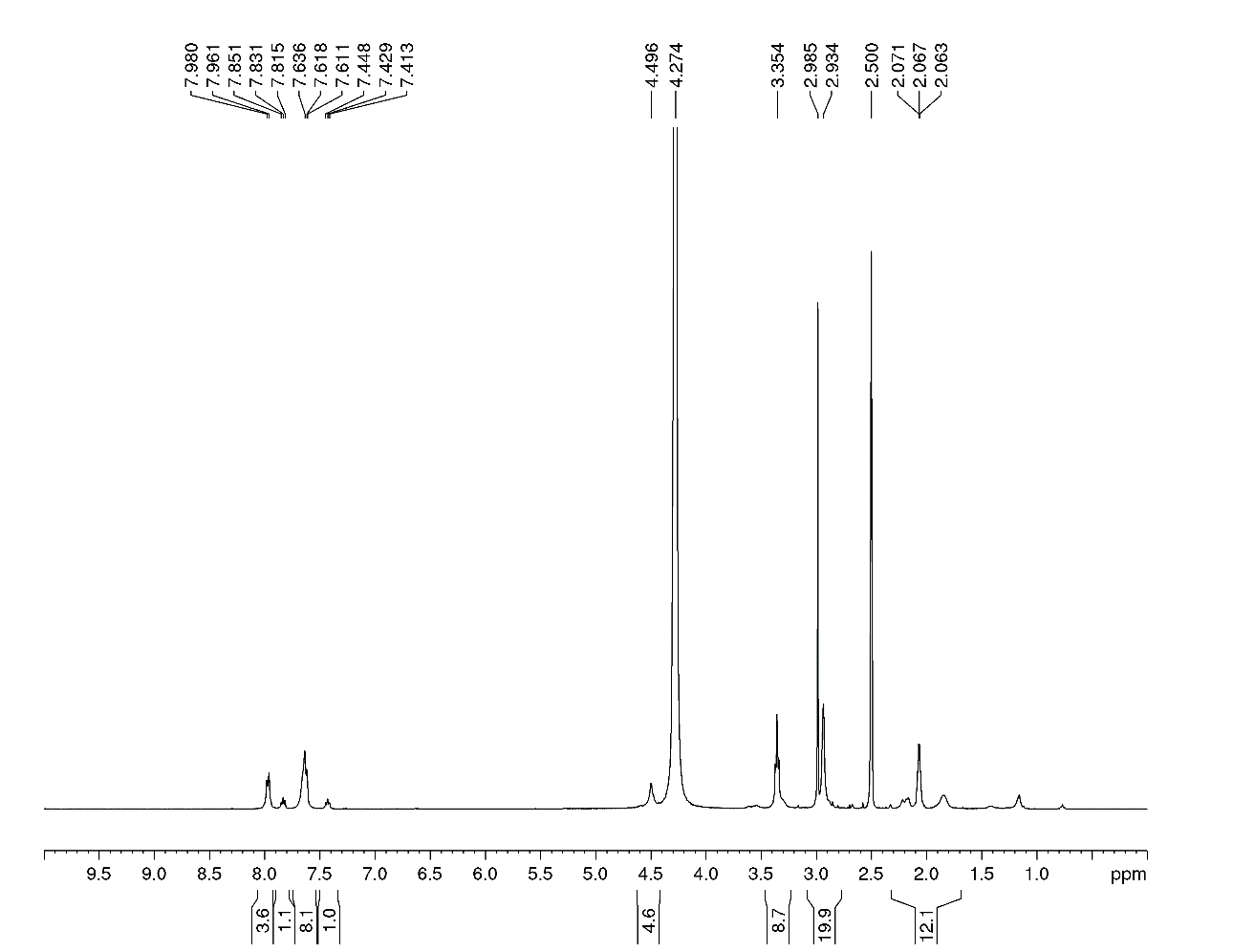


**Figure S16.** ^1^H NMR (400 MHz, DMSO-*d_6_*: D_2_O, 5:1 *v*/*v*) of *p*-C_4_.


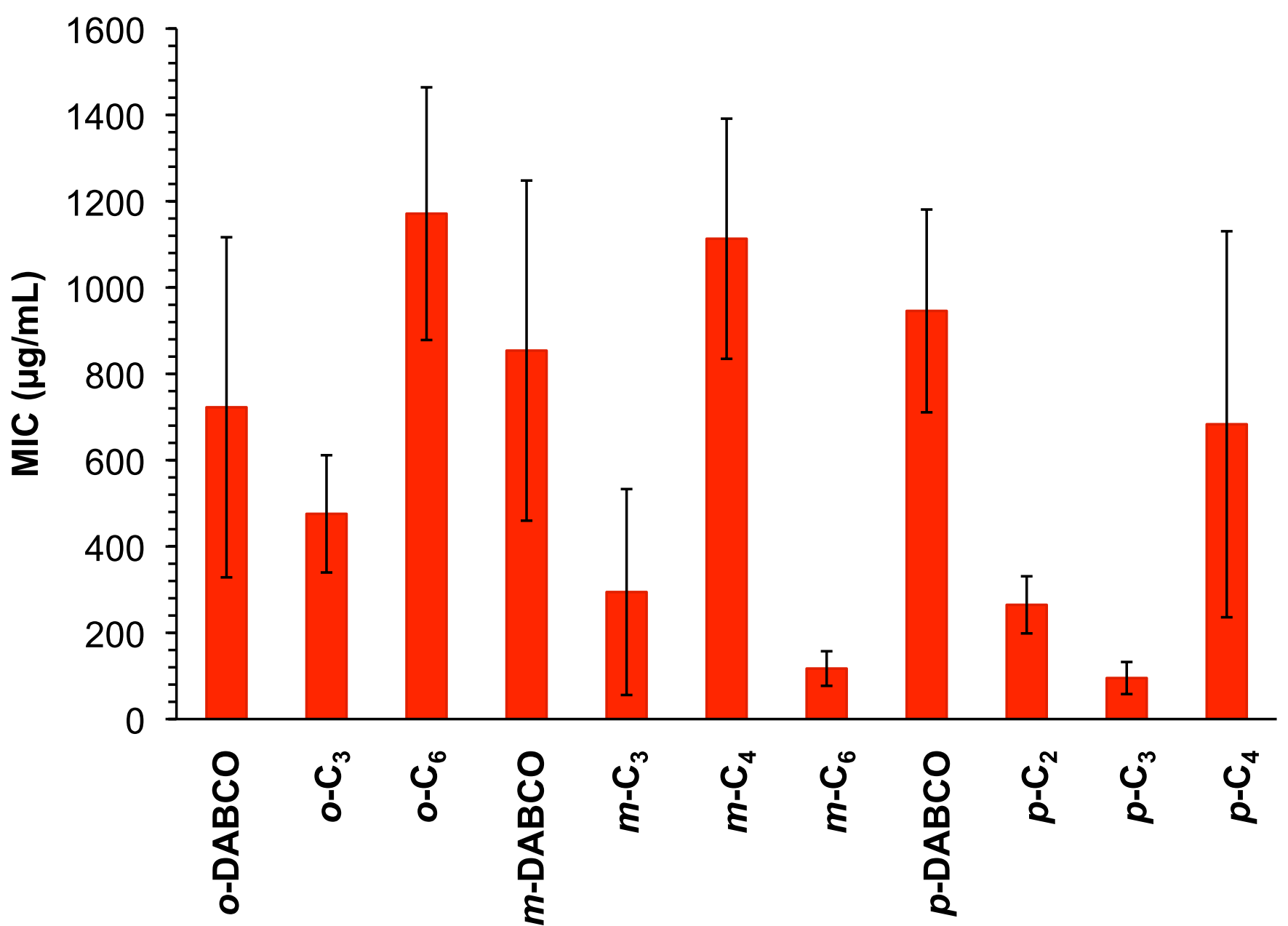


**Figure S17.** Minimum inhibitory concentrations (MIC), expressed in μg/mL, of ionene suspensions for inhibition of bacterial growth.


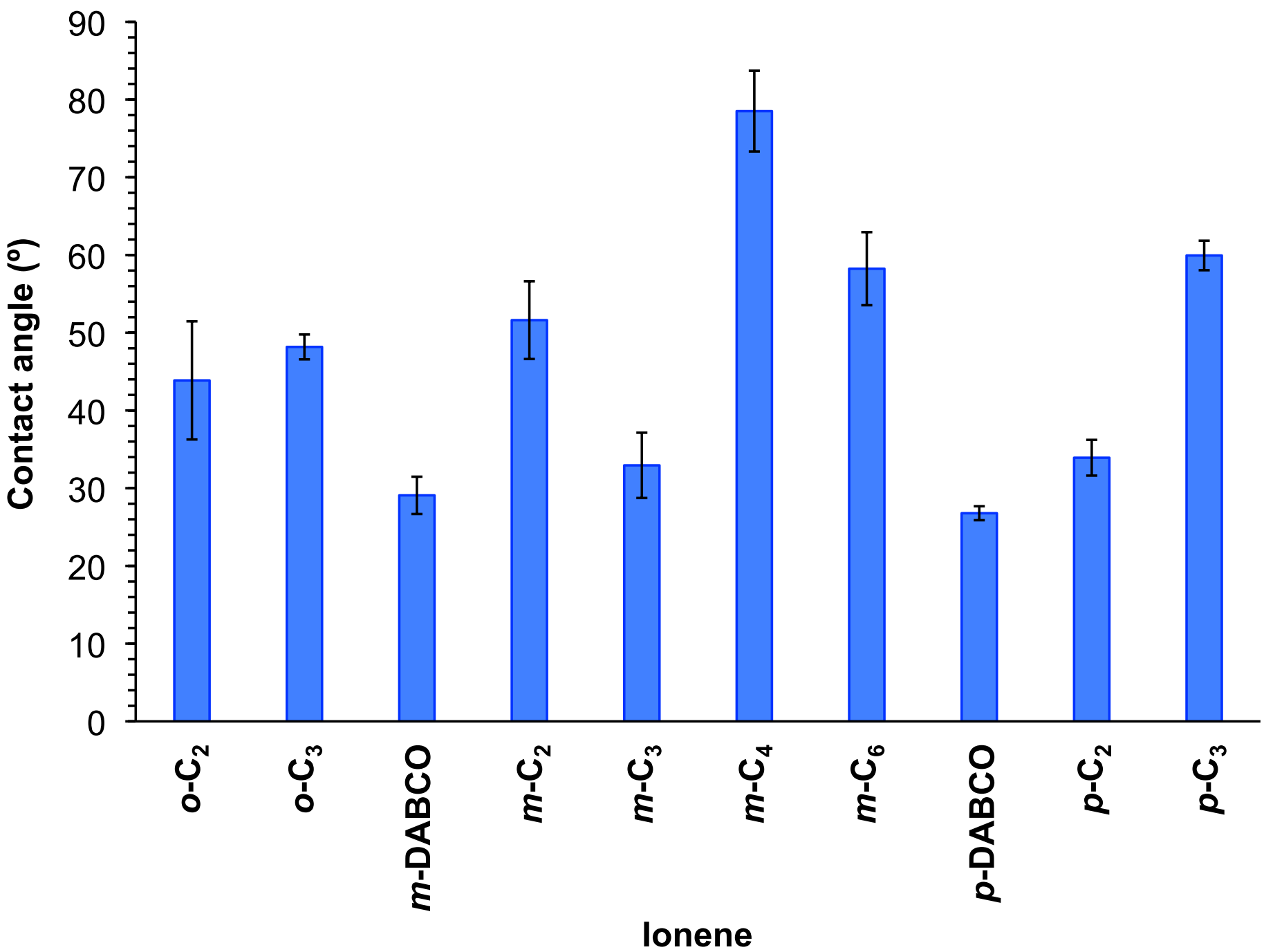


**Figure S18.** Static contact angle for films prepared with the different ionenes as described in the Experimental Section. Reliable measurements were not obtained for samples prepared with *o*-DABCO (too hydrophilic), *p*-C4 (too hydrophilic) and *o*-C_6_ (inhomogeneous film).


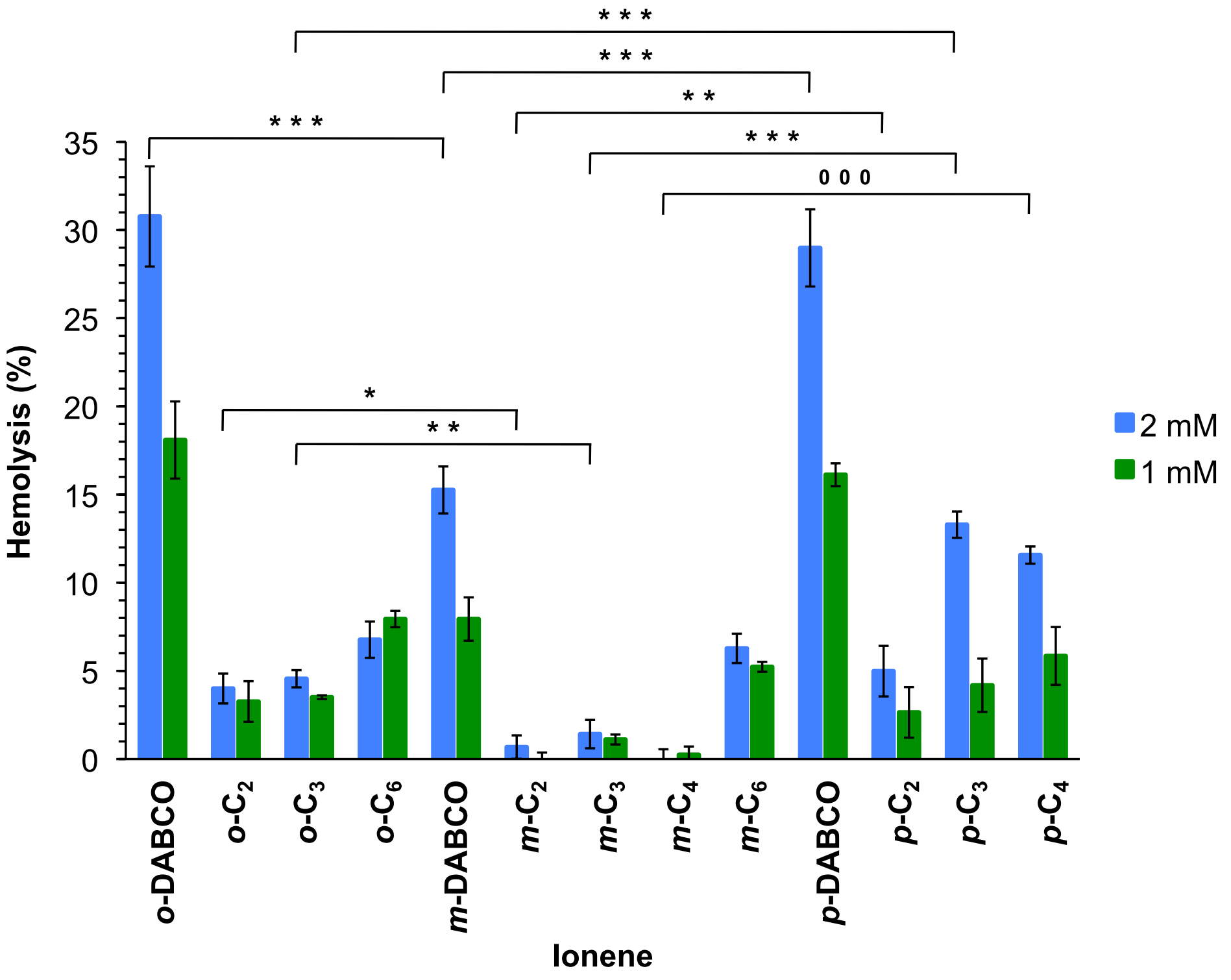


**Figure S19.** Mean values of hemolysis from at least three replicates with SD and ANOVA with Bonferroni comparison or or unpaired *t*-test. Comparison between different linkers. * Indicates *p* < 0.05; ** indicates *p* < 0.01; ***^/000^ indicates *p* < 0.001.


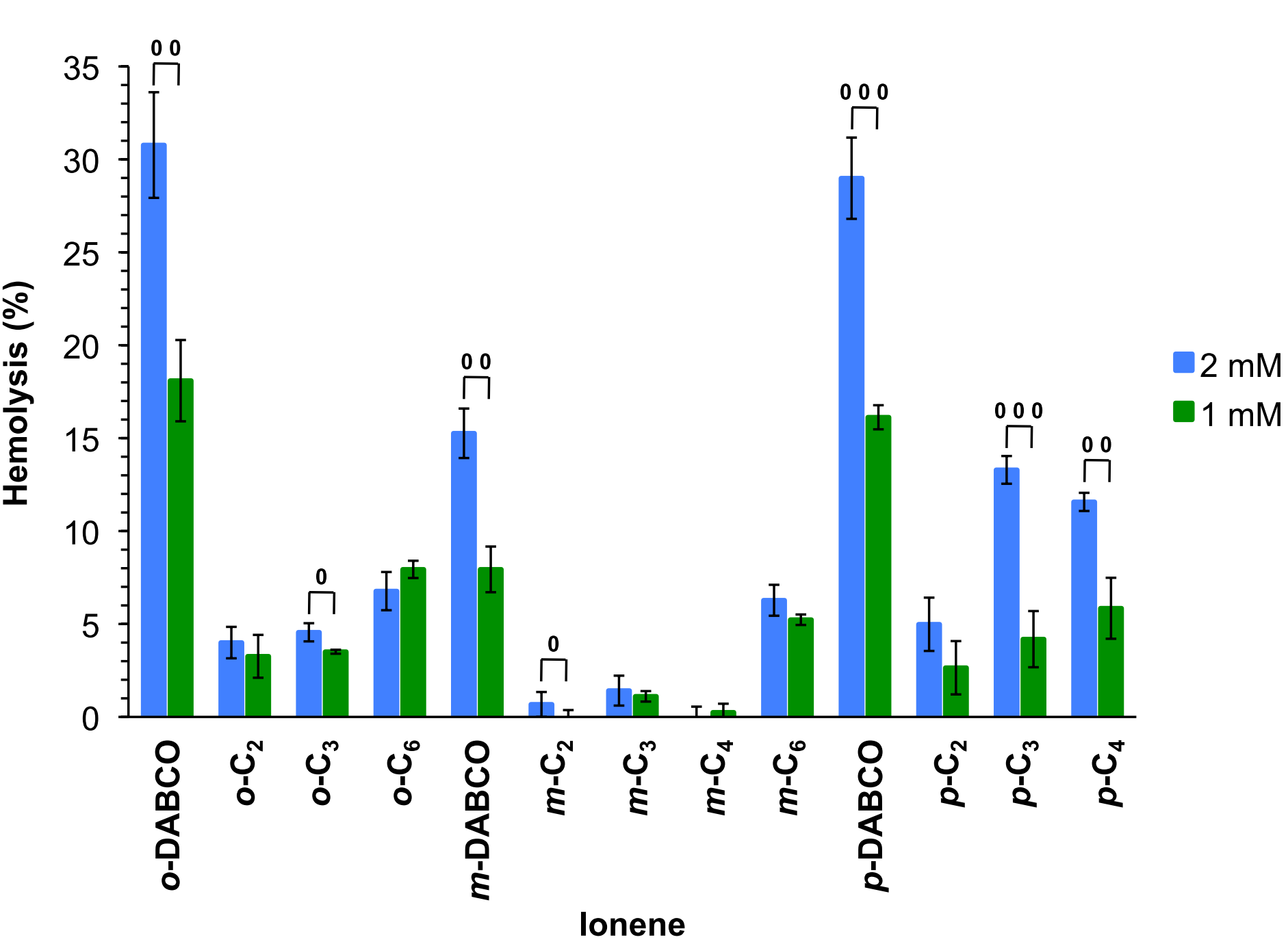


**Figure S20.** Mean values of hemolysis from at least three replicates with SD including significance according to unpaired *t*-test. Comparison between 2 and 1 mmol/L. ^0^ Indicates *p* < 0.05; ^00^ indicates *p* < 0.01; ^000^ indicates *p* < 0.001.
